# Hydro-alcoholic extract of the root of *Prangos ferulacea *(L.) Lindl can improve serum glucose and lipids in alloxan-induced diabetic rats

**Published:** 2012

**Authors:** Najme Kafash Farkhad, Farah Farokhi, Amir Tukmacki, Khosro Soltani band

**Affiliations:** 1 *Department of Biology, Faculty of Science, Urmia University, Urmia, I. R. Iran*; 2 *Department of Biology, Faculty of Science, Urmia University, Urmia, I. R. Iran*; 3 *Department of Artemias Rresearch, Faculty of Veterinary, Urmia University, Urmia, I. R. Iran*

**Keywords:** Diabetes Mellitus, Hypoglycemic, Hypolipidemic, Lipid profile, *Prangos ferulacea *(L.) Lindl

## Abstract

**Objectives:** Diabetes mellitus manifests itself in a wide variety of complications and the symptoms of this disease are multifactorial. Previous studies proved that this disease is directly related to hyperglycemia and hyperlipidemia. The aim of this study was to investigate the hypoglycemic and hypolipidemic effects of hydroalcoholic extract of *Prangos frulacea* (L.) Lindl in alloxan-induced diabetic rats.

**Materials and Methods: **Forty female Wistar rats with body weight of 200±20 g were randomly divided into five groups with eight rats per group. Diabetes was induced in rats by alloxan monohydrate at dose of 120 mg/kg body weight (BW) injected intraperitoneally. Hydro-alcoholic extract of the root and leaves with stems of *P. frulacea *at 100 mg/kg BW were given orally to diabetic rats daily for 4 weeks.

**Result: **Diabetic rats (D) exhibited a significant (p<0.05) increase in the levels of the serum glucose, Total Cholesterol (TC), Triglycerides (TG), and LDL in comparison with the control group whereas their BW and serum HDL levels were decreased. In diabetic rats treated by root extract of *P. frulacea, *these parameters were reversed to the normal levels compared with diabetic group.

**Conclusion:** According to the results obtained, it was concluded that Root´s hydro-alcoholic extract of *P. frulacea *can be used in diabetics for the purpose of glucose and lipid profile reduction.

## Introduction

Diabetes mellitus is the name given to a group of disorders with different etiologies. It is characterized by disarrangements in carbohydrates, proteins, and fat metabolism. It is caused by the complete or relative insufficiency of insulin secretion and/or insulin action (Milagro et al., 2000 ▶). Recently, medicinal values of various plants extracts have been studied by many scientists in the field of diabetic research (Devi et al., 2010 ▶).

Poorly controlled blood glucose is believed to be the most important factor in the development of diabetic complications in both type 1 and type 2 diabetes. Diabetes is characterized by symptoms such as weakness, polyurea, excessive thirst as well as ketonemia, ketonuria, and ketosis due to altered metabolism of lipids and proteins (Devi et al., 2010 ▶). In a diabetic condition, increased serum lipids are due to the increased lipolysis of adipose tissue, and thereby cause abnormal lipoprotein concentration (Mc Kenney, 2001 ▶). 

Throughout the world many plants are used in traditional medicine to treat diabetes mellitus and they represent valuable alternatives for the control of this disease. The ethnobotanical information reports about 800 plants that may possess antidiabetic potential (Alarcon et al., 1998 ▶). More than 400 plants with glucose-lowering effects are known. Among these plants, some have been reported to possess hypoglycemic effects (Prince et al., 1998 ▶) and some hypolipidemic effects (Mhaskar et al., 2000 ▶). However, there is little information about plants with both hypoglycemic and hypolipidemic effects (Nagarajan et al., 2005 ▶).

In this aspect, *P. ferulacea *from Appiacea (umblifera) family grows widely in southern and many other regions of Iran and used in Iranian herbal medicine for gastrointestinal disorders (Coruh et al., 2007 ▶), but it seems it has a hypoglycemic effect on diabetic patients. Unfortunately, there are no sicientific available reports for this claim. Therefore, we undertook the present investigation to evaluate the hypoglycemic and hypolipidemic effects of this plant in alloxan-induced diabetic rats.

## Material and Methods

**Chemicals and drugs**

Alloxan monohydrate and chloroform were purchased from Sigma Chemicals, Germany. Insulin NPH from Exir Pharmaceutical Company, normal saline from Iran Daroupakhsh Company, ethanol from Pakdis Company, Iran and other materials were purchased from Merk Company, Germany.

**Plant material**

Fresh, green *P. ferulacea *plants were collected from the Shahidan Mountains of West Azerbaijan in northwest of Iran in frontier localities between Iran and Turkey in May 2010 and authenticated by a professor from the Department of Biology at Urmia University. The samples (roots separately, and green leaf and stems had weight rate of 1:1) were dried in shadow for seven days.

**Preparation of extracts**

Collected samples were dried and ground by an electrical mill. One hundred grams of both powder samples were added to 1000 ml of alcohol. First, ethanol 96% was used and after 24 h both solutions were filtered. In the second step, ethanol 70% was added to the remained dry materials. After 24 h, solutions were filtered and then both filtered solutions were mixed together and then evaporated repeatedly to half the first volume by rotary evaporator in 50º C and 70 rpm. Concentrated extracts were dried on water bath at 40º C temperature to yield 6% w/w dry extract. For the preparation of injected extract, this powder was solved in specific volume of normal saline (Larkins et al., 2004 ▶).

**Preparation of diabetic rats**

Alloxan monohydrate dissolved in saline was injected to rats intraperitoneally at dose of 120 mg/kg body weight (BW). After a fortnight, rats with marked hyperglycemia (serum glucose more than 200 mg/dl) were selected and used for the study (Kazerooni et al., 2006 ▶). In our experiment about 10-15% of rats after alloxan injection were died but nearly about 60-70% of other rats have shown diabetic sympotomes such as polyphagia,polyurea and markedly hyperglycemia.

**Experimental design**

Forty female Wistar rats with BW of 200±20 g were purchased from Pasteur Institute, Iran, and were kept in animal houses of Urmia University. They were kept at 20±5º C, relative humidity of 30±5%, and light/dark cycle for 12 h. All animals were fed with rodent pellet diet (purchased from Pars Company, Karaj) and water was allowed ad libitum under strict hygienic conditions. These rats were randomly divided into five groups with eight rats per group as follows: group 1 (C: controlled group) were administrated 0.5 ml saline, group 2 (D: untreated diabetic rats), group 3 (D+S1) diabetic rats receiving roots hydro-alcoholic extract of *P. ferulacea *at 100 mg/kg BW in saline, group 4 (D+S2) diabetic rats receiving hydro-alcoholic extract of leaves and stems of *P. ferulacea *at 100 mg/kg BW in saline, and group 5 (D+S3) diabetic rats receiving insulin NPH at 1 IU/kg. Treatments periods were four weeks and all extracts were given orally in rats by intra-gastric tube.

**Biochemical estimation**

At the end of the experiment (In the 28^th^ day), the rats were weighed, anesthetized using diethyl ether and serum samples from all of them were collected for estimation of biochemical parameters, serum glucose (GOD-POD method), cholesterol (CHOD-PAP method), triglycerides (GPO-Triender method), HDL (High lipoprotein density), and LDL (Low lipoprotein density) (Kar et al., 2003). In these methods, enzymatic kits (Merk Company, Germany) were used. Serum glucose and body weight (BW) for all experimental groups was measured three times (0, 2, and 4 weeks).

**Statistical analysis**

All values are expressed as Mean±SEM. The differences were compared using one way analysis of variance (ANOVA) followed by Tukey's multiple comparison tests. For all analyses, p-values<0.05 were considered statistically significant.

## Results

Effects of *P. ferulacea *extracts on body weight (BW) of experimental groups are shown in the following tables: Table 1 illustrates the variations in BW in normal control, diabetic control, and diabetic treatment groups in three times (at the start of experiment, 2, and 4 weeks after alloxan injection). Alloxan significantly (p<0.01) reduced the body weight of diabetic rats compared with the controls, which gained significant weight. After 2 weeks, only insulin (p<0.01) and hydroalcoholic extract of roots of *P. ferulacea* (p<0.05) treated diabetic rats showed significant increase in BW. Although the extract of stems & leaves of *P. ferulacea *at 100 mg/kg BW ameliorated this weight loss after 4 weeks, the extract of the roots at 100 mg/kg demonstrated a significant beneficial effect when compared with the reference drug Insulin.

**Table 1:** Body weight of experimental groups in 0, 14, and 28 days after the start of the experiment

**Treatment Time**	**Control**	**Diabetic**	**D+S1**	**D+S2**	**D+S3**
At the start of the experiment (day 0)	207.4±11.3	204±10.5	210±13.2	213.1±10.8	205.6±15.3
After 14 days	221±17.3**	147.1±14.1	184.2±11*	154.1±10.3	209.4±9.8**
After 28 days	254.6±21.4**	139.9±19.2	200.1±26.4**	143.2±17.2	211.4±23.4**

* p<0.05 and

** p<0.01, indicate significant changes compared to diabetic group. D+S1: diabetic rats treated with hydroalcoholic extract of *P. ferulacea *roots (100 mg/kg), D+S2: diabetic rats treated with hydroalcoholic extract of stems & leaves of *P. ferulacea* (100 mg/kg), D+S3: diabetic rats treated with insulin NPH (1 IU/kg).

**Figure 1:**
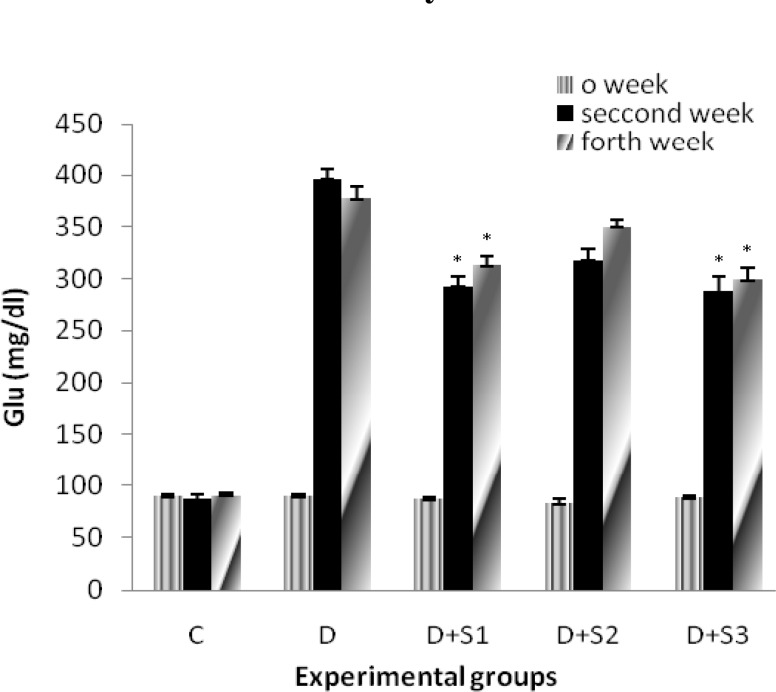
Effect of hydroalcoholic extract of *P. ferulacea *on serum glucose levels in experimental groups on 0, 14, and 28 days. All values are expressed as Mean±SEM (n=8). Statistical comparisons between each group were carried out by one way ANOVA followed by Tukey´s multiple comparison tests.

Effects of *P. ferulacea *extracts on glucose levels of experimental groups are as follows: As shown in Figure 1, at the start of the experiment, there was no significant difference in blood glucose between experimental groups and all of the groups had normal rates of glucose (83.4±7.5). Alloxan injection caused significant increase in blood glucose in D group after 2 and 4 weeks (396.6±19.1, 383.2±13.2) in comparison with C group. Treatment with hydroalcoholic extract of roots of *P. ferulacea *after 2 and 4 weeks caused significant decrease in this parameter (291.33±14, 309.1±9.6) in comparison with D group. In addition, in insulin treated group, this decrease was significant (280.1±8.1, 298.1±11). Extract of stems & leaves of *P. ferulacea *had no significant effects on serum glucose levels and glucose levels in this group were close to the diabetic rats after 2 and 4 weeks (319.3±14.1, 350±11.2).

**Figure 2:**
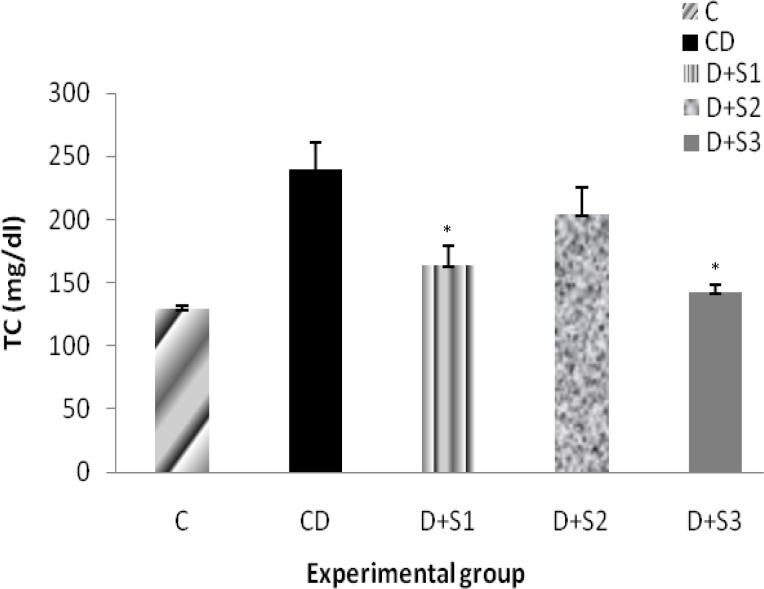
Effect of hydroalcoholic extract of *P. ferulacea *on serum Total cholestrol (TC) levels in experimental groups in 28 days. All values are expressed as Mean±SEM (n=8). Statistical comparisons between each group were carried out by one way ANOVA followed by Tukey´s multiple comparison tests.

Effects of *P. ferulacea *extracts on total cholestrol (TC) of experimental groups are as follow: As shown in Figure 2, serum cholestrol levels were significantly (p<0.05) higher in diabetic rats (249.7±6.9) as compared to the control rats (130.2±4.1). TC in rats treated with hydro-alcoholic extract of the roots of *P. ferulacea *(163.5±8.1) and with insulin (140.1±3.9) significantly decreased as compared to untreated diabetic rats. There were no significant difference between D+S2 and D group, and TC in D+S2 was in 207±1.4 mg/dl rates.

**Figure 3:**
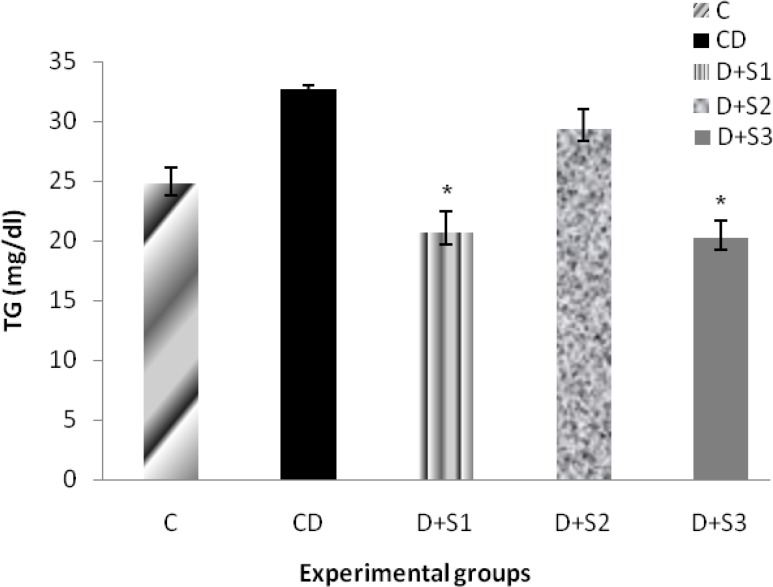
Effect of hydroalcoholic extract of *P. ferulacea *on serum TG (Triglycerides) levels in experimental groups in 28 days. All values are expressed as Mean±SEM (n=8). Statistical comparisons between each group were carried out by one way ANOVA followed by Tukey´s multiple comparison tests.

Effects of *P. ferulacea *extracts on triglycerides levels (TG) of experimental groups are as follow: Analysis of variance results showed that diabetes induction caused significant increase in triglyceride levels of diabetic rats (33.4±1.7) in comparison with the control group (24.8±1.2). Treatment with hydro-alcoholic extract of the roots of *P. ferulacea *after 4 weeks caused significant (p<0.05) decrease in TG (21.8±1.1) and in D+S3 group insulin significantly (p<0.001) have decreased this parameter (19.2±1.3) as shown in Figure 3. In D+S2 group, TG levels were close to the D group (29.4±1.6).

**Figure 4:**
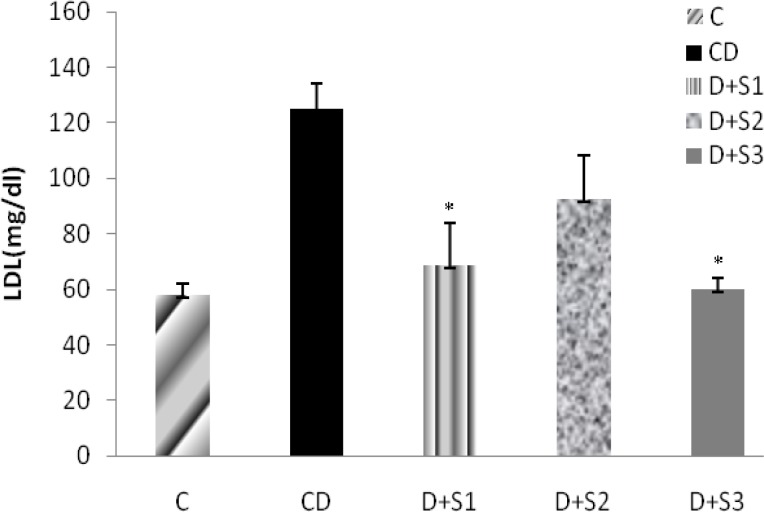
Effect of hydroalcoholic extract of *P. ferulacea *on serum LDL (Low Lipoprotein Density) levels in experimental groups in 28 days. All values are expressed as Mean±SEM (n=8). Statistical comparisons between each group were carried out by one way ANOVA followed by Tukey´s multiple comparison tests.

Effects of *P. ferulacea *extracts on Low Lipoprotein Density (LDL) levels of experimental groups were as follow: According to Figure 4, alloxan injection significantly (p<0.05) increased LDL levels in diabetic rats (124.6±7.8) as compared to the controls (58.1±3.8). Four weeks treatment with hydro-alcoholic extract of *P. ferulacea *roots and insulin caused decrease in serum LDL levels (64.1±3.1 & 58.8±2.1). Even though in D+S2 group there was increase in serum LDL levels, but this improvement was not significant (90.2±8.3).

**Figure 5:**
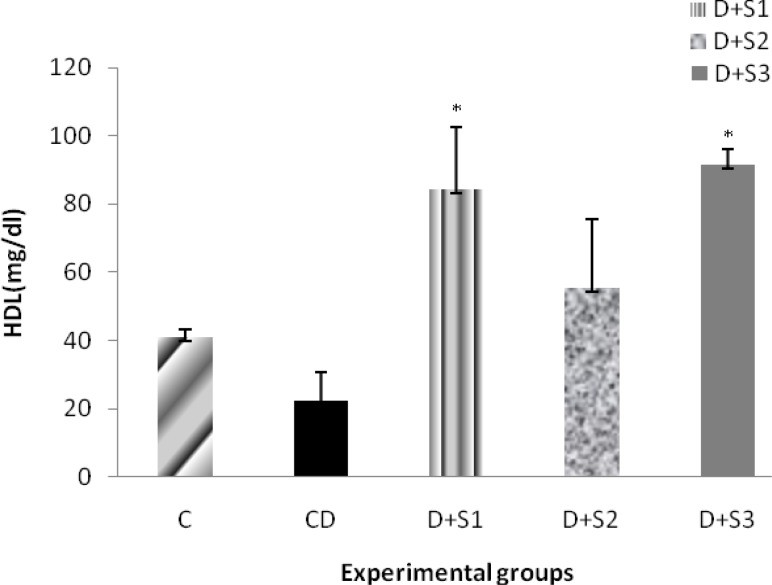
Effect of hydroalcoholic extract of *P. ferulacea *on serum HDL (High Lipoprotein Density) levels in experimental groups in 28 days. All values are expressed as Mean±SEM (n=8). Statistical comparisons between each group were carried out by one way ANOVA followed by Tukey´s multiple comparison tests.

Effects of *P. ferulacea *extracts on High Lipoprotein Density (HDL) levels of experimental groups are as follow: As shown in Figure 5, serum HDL was significantly (p<0.05) lower in diabetic rats (25.2±6.1) compared to the control rats (41.1±2.1). Analysis of variance results showed that the mean difference between the D+S1 (84.1±9.4) and D+S3 (87.1±2.5) groups in comparison with the D group is significant (p<0.05), which indicates that the root extract and insulin have increased HDL level in diabetic rats. Extract of the stems and leaves of *P. ferulacea *had no significant effects on serum HDL levels. HDL levels in this group were at 51.9±4.2 mg/dl.

* p<0.05 in comparison with diabetic values.

C: control group; D: diabetic group; D+S1: diabetic rats treated with hydroalcoholic extract of the roots of *P. ferulacea *(100 mg/kg); D+S2: diabetic rats treated with hydroalcoholic extract of the stems and leaves of *P. ferulacea *(100 mg/kg); D+S3: diabetic rats treated with insulin NPH (1 IU/kg).

* p<0.05 in comparison with diabetic values.

C: control group; D: diabetic group; D+S1: diabetic rats treated with hydroalcoholic extract of the roots of *P. ferulacea *(100 mg/kg); D+S2: diabetic rats treated with hydroalcoholic extract of the stems and leaves of *P. ferulacea *(100 mg/kg); D+S3: diabetic rats treated with insulin NPH (1 IU/kg).

* p<0.05 in comparison with diabetic values.

C: control group; D: diabetic group, D+S1: diabetic rats treated with hydroalcoholic extract of the roots of *P. ferulacea *(100 mg/kg); D+S2: diabetic rats treated with hydroalcoholic extract of the stems and leaves *P. ferulacea *(100 mg/kg); D+S3: diabetic rats treated with insulin NPH (1 IU/kg).

* p<0.05 in comparison with diabetic values.

C: control group; D: diabetic group; D+S1: diabetic rats treated with hydroalcoholic extract of the roots of *P. ferulacea *(100 mg/kg); D+S2: diabetic rats treated with hydroalcoholic extract of the stems and leaves of *P. ferulacea *(100 mg/kg); D+S3: diabetic rats treated with insulin NPH (1 IU/kg).

* p<0.05 in comparison with diabetic values.

C: control group; D: diabetic group; D+S1: diabetic rats treated with hydroalcoholic extract of the roots of *P. ferulacea *(100 mg/kg); D+S2: diabetic rats treated with hydroalcoholic extract of the stems and leaves of *P. ferulacea *(100 mg/kg); D+S3: diabetic rats treated with insulin NPH (1 IU/kg).

## Discussion

Hyperglycemia and hyperlipidemia are important characteristics of diabetes mellitus, an endocrine disorder which is one of the most common chronic diseases worldwide (Milagro et al., 2000 ▶). The present study was designed to investigate the hypoglycemic and hypolipidemic effects of hydroalcoholic extract of *P. ferulacea.*

In our experiment, we observed higher levels of glucose and lipids in alloxan diabetic rats that correlates with the previous research findings (Byung et al., 2001 ▶). D+S1 and D+S3 groups showed a significant decrease in these parameters as comparison with the D group, but in the D+S2 group this decrease was not significant.

Diabetes mellitus is one of the most common metabolic diseases and dearangements in lipid metabolism (Milagro et al., 2000 ▶). The marked increase in serum triglycerides and cholesterol observed in untreated diabetic rats is in agreement with the findings of Nikkila and Kekki (1973) ▶. 

Under normal circumstances insulin activates lipoprotein lipase enzyme and hydrolyses triglycerides. Insulin deficiency results in failure to activate the enzymes thereby causing hypertriglyceridemia. The significant control of serum lipids in the diabetic rats treated with hydroalcoholic extract of the roots (D+S1) may be directly attributed to improvements in insulin levels. As previous studies reported (Coruh et al., 2007 ▶), roots of *P. ferulacea *have a natural antioxidant, umbelliferone (Ramesh et al., 2005 ▶), that can elevate insulin secretion from β pancreatic cells. Therefore, hypoglycemic and hypolipidemic effects of the hydro-alcoholic extract of the roots of *P. ferulacea *may be related to this component. In D+S2 group, after treatment with extract, elevated glucose and lipid was partially ameliorated but this improvement was not significant. One probable reason is that in absence of umbelliferone, decreased insulin secretion in this group, have not been reversed to the normal levels, so hyperglycemic and hyperlipidemic conditions induced by diabetes, were not improved. As we know that umbelliferone is soluted in fat (Ramesh et al.,2005 ▶) and alcohol can solutede the fat, we can conclude that alcoholic part of this hydroalcoholic extract have shown this effective results. In addition, there may exist some components in leaves of *P. ferulacea *that can neutralize the antioxidant properties of stem´s components or vice versa, but more studies are needed.

In diabetic rats, decreased body weight (BW) was observed. This indicates the polyphagic condition and loss of weight due to excessive breakdown of tissue proteins (Hakim et al., 1997 ▶). The decrease in body weight in diabetic rats could be due to dehydration and catabolism of fats and proteins (Kamalakkannan et al., 2006 ▶). Our results were in agreement with those previous findings and as it is shown in table 1, in diabetic rats (D group) BW was decreased significantly and treatment with hydroalcoholic extract of roots of *P. ferulacea *(D+S1 group) and insulin (D+S3 group) after 4 weeks can increase BW and reverse this parameter close to the normal group (C group).

Elevation of plasma lipid concentration in diabetes is well documented (Chase et al., 1976 ▶; Shirwaikar et al., 2004). Increased catabolic reactions leading to muscle wasting might also be the cause for the reduced weight gain by diabetic rats (Kamalakkannan et al., 2006 ▶). In insulin deficient diabetics, the plasma free fatty acid concentration is elevated as a result of increased free fatty acid outflow from fat depots, where the balance of the free fatty acid esterification–triglyceride lipolysis cycle is displaced in favor of lipolysis. Increased serum cholesterol in diabetic rats of the present experiments may be due to diminishing the clearance of cholestrol from blood (Shirwaikar et al., 2004). 

Plasma LDL can undergo reuptake in the liver via specific receptors and get cleared from the circulation. This increase in LDL concentration may be due to defective receptors for LDL (Rajasekaran et al., 2006 ▶). HDL can be protected by reversing cholesterol transport, inhibiting the oxidation of LDL, and by neutralizing the atherogeneic effects of oxidized LDL (Bhagavan, 2002 ▶). HDL helps to scavenge cholesterol from extra hepatic tissues and decreased HDL can contribute to the increased cholesterol levels. A greater increase of LDL may cause a greater decrease of HDL as there is a reciprocal relation between the concentration of LDL and HDL (Georg et al., 2008 ▶). 

Modern medicines such as biguanides, sulphonylureas, and thiozolidinediones are available for treating diabetes. However, they also have side effects and cannot give a long term glycemic control (Devi et al., 2010 ▶). Alternative medicines particularly herbal medicines are available for the treatment of diabetes. Common advantages of herbal medicines are their effectiveness, safety, affordability, and acceptability (Sayed et al., 2011 ▶). Medicinal plants are a rich source for natural products and their products have been widely used for treatment of diabetes all around the world with less known scientific basis of their functioning (Sayd et al., 2007 ▶).

The genus of *Prangos *with the common Persian name of Jashir includes 15 species which are growing widely in many regions of Iran. Previous studies proved that antioxidant properties of this plant are more than α-tocopherole (vitamine E). Some constituents of this plant were detected to be flavonoids, coumarines, alkaloids, terpenoids, and umbelliferon (Coruh et al., 2007 ▶). As reported earlier, all of these components have an antioxidant effect that is effective against oxidative stress (Coruh et al., 2007 ▶).

Oxidative stress generated by hyperglycemia and hyperlipidemia are regarded as important mediators of diabetic complications (Giugliano et al., 1996 ▶). The presence of free radicals and the simultaneous decline of antioxidant defense mechanisms observed in diabetic patients could promote the development of diabetic complications (Byung et al., 2001 ▶). It was proved that high levels of flavenoid and polyphenolic compounds, especially umbelliferone, are directly related to normal amount of glucose and lipid levels (Byung et al., 2001 ▶). As indicated in the present results, lipid concentration and glucose levels in extract of the roots of *P. ferulacea *treated rats (D+S1 group) were reversed to the normal levels. That can prove antihyperglycemic and antihyperlipidemic effects of this extract.

In summary, these results suggest that there are increased glucose levels and concentration of lipids in diabetic rats. Hydroalcoholic extract of the roots of *P. ferulacea *showed hypoglycemic and hypolipidemic activity and reduced the level of glucose and lipids which was elevated in diabetic control rats. However, hydroalcoholic extract of the stems and leaves of *P. ferulacea *had no significant effect on those parameters. 
